# The long-tail effect of the COVID-19 lockdown on Italians’ quality of life, sleep and physical activity

**DOI:** 10.1038/s41597-022-01376-5

**Published:** 2022-05-31

**Authors:** Michela Natilli, Alessio Rossi, Athos Trecroci, Luca Cavaggioni, Giampiero Merati, Damiano Formenti

**Affiliations:** 1 National Research Council (CNR), Institute of Information Science and Technologies (ISTI), Pisa, Italy; 2 Department of Computer Science, University of Pisa, Pisa, Italy; 3 Department of Biomedical Science for Health, University of Milan, Milan, Italy; 4 Department of Endocrine and Metabolic Diseases, Obesity Unit and Laboratory of Nutrition and Obesity Research, IRCCS Istituto Auxologico Italiano, Milan, Italy; 5 Department of Biotechnology and Life Sciences, University of Insubria, Varese, Italy; 6 IRCCS Fondazione Don Carlo Gnocchi, Milan, Italy

**Keywords:** Quality of life, Population screening

## Abstract

From March 2020 to May 2021, several lockdown periods caused by the COVID-19 pandemic have limited people’s usual activities and mobility in Italy, as well as around the world. These unprecedented confinement measures dramatically modified citizens’ daily lifestyles and behaviours. However, with the advent of summer 2021 and thanks to the vaccination campaign that significantly prevents serious illness and death, and reduces the risk of contagion, all the Italian regions finally returned to regular behaviours and routines. Anyhow, it is unclear if there is a long-tail effect on people’s quality of life, sleep- and physical activity-related behaviours. Thanks to the dataset described in this paper, it will be possible to obtain accurate insights of the changes induced by the lockdown period in the Italians’ health that will permit to provide practical suggestions at local, regional, and state institutions and companies to improve infrastructures and services that could be beneficial to Italians’ well being.

## Abstract (summary)

**Table:** 

Measurement(s)	Quality of Life • Overall Sleep Quality Rating • Physical Activity Measurement
Technology Type(s)	SF-36 • Overall Sleep Quality Rating • International Physical Activity Questionnaire (August 2002) Short Last 7 Days Self-Administered Format
Sample Characteristic - Organism	Homo sapiens
Sample Characteristic - Environment	questionnaire

## Background & Summary

The World Health Organization (WHO) announced on 11 March 2020 that the new coronavirus disease (i.e., COVID-19) could be classified as a pandemic due to its high contagion rate and the overall worldwide mobility. In an attempt to restrict and limit the spread of the disease, governments introduced restriction and confinement strategies that immediately affect peoples’ routines and usual activities^1^. Italy was one of the first European countries subjected to the pandemic state. On February 21th, 2020, the first cluster of COVID-19 cases was detected in Lombardy (Italy). Since then, the Italian government adopted an increasing number of measures aimed at promoting social distancing, such as closure of bar and restaurants, non-essential business and industries, school and universities, thus effectively inducing a national state of lockdown^2^. From March 2020 to May 2021, several lockdown periods have limited, with varying degrees of severity, the people’s usual activities and mobility in Italy, as well as around the world.

These unprecedented confinement measures, together with the COVID-related consequences, dramatically modified the citizens’ daily lifestyle and behaviors^3^. Besides the serious economic damages, social and psychological negative effects of social distancing and isolation have been quickly recognised by the scientific community^4,5^ and government itself. Since the COVID-19 diffusion, several studies demonstrated that the lockdown periods negatively affected mental health^6^, sleep-^7–10^ and physical activity-related behaviors^11^, overall contributing to reduced well-being and quality of life^12–14^. This picture illustrates the immediate dramatic consequence of home-confinement, that Italian and other governments worldwide adopted to limit the COVID-19 diffusion and preserve citizens’ health. To the best of the authors’ knowledge, the majority of the literature addressed the consequences of lockdowns on wellbeing by gathering psychological and behavioral data throughout lockdown periods employing a retrospective comparison between before and directly during COVID-19 lockdowns. This pre-during comparison design captured only half of the complete picture reflecting the potential consequences of lockdowns. However, with the advent of summer 2021, thanks also to the vaccination campaign that significantly prevented the progression to serious illness and death, and reduced the risk of contagion, all the Italian regions finally returned to regular moving behaviors and routines. In spite of this, it is plausible that the potential consequences of lockdowns are still persistent within the Italian citizens, and whether people’s quality of life, sleep- and physical activity-related behaviors have returned to the pre-lockdowns level is still unclear.

To illustrate this second half of the complete picture, we designed a project aimed at gathering subjective data assessing quality of life, sleep- and physical activity-related behaviors. The project includes the collection and curation of a database derived from an online survey shared between November and December 2021, in which questionnaires quantifying quality of life, sleep, and physical activity were proposed referring to two different moments: before COVID-19 quarantine (i.e., November 2019) and the same time period in which the survey was filled in. Participants countrywide reported their subjective data of these two reference time periods through a smartphone or personal computer, developed specifically for this purpose. Through this methodology, the project aimed to provide pre-post lockdowns comparison data to track whether people’s quality of life, sleep- and physical activity-related behaviors have effectively returned to the pre-lockdowns level. As the lockdown periods might have a different effect on each participant, depending on their specific context, we have also collected contextual information, such as socioeconomic status, living space, employment changes. These data may be useful to investigate aspects of the psychological and behavioral impact of the COVID-19 lockdowns. Researchers and authorities may explore these data to quantify the population’s subjective perception of their general wellbeing, in relation to certain context variables.

## Methods

In order to collect data on the Italians perception on their physical activity, quality of sleep, and quality of life before and after the first Italian general lockdown (9th March 2020), an online questionnaire was created and administered using Qualtrics® Survey Platform (https://www.qualtrics.com/).

The data gathering was conducted between November 1st 2021 and January 12th 2022 in order to compare similar seasonal periods before and after the lockdown, avoiding in this way any possible influence of the season on physical activity habits and quality of life in general.

The research protocol for this study was approved by the bioethical committee of the University of Pisa (27/2021) and the ethical commitee of the European Community’s H2020 Program SoBigData++ research infrastructure (BOEL20211018RP). Before filling in the questionnaire, participants were asked to read and approve an informed consent for the use of the personal data. The consent document deeply explained the project’s aims, methods, possible risks, and benefits of the study. The personal data (e.g., Name, Surname and personal contact) were not recorded in order to guarantee the privacy of the respondents. Participants provided consent to share survey data.

### Study design

A cross-sectional study design (with some retrospective questions) was used. People residing in Italy aged between 18 and 60 were recruited via institutional e-mails (i.e., University of Pisa, University of Milan, and University of Insubria) and by social networks (i.e., Twitter, Linkedin, Instagram and Facebook). Since we did not know the population, we opted for a non-probabilistic approach using mainly social media to spread the survey. In details: the University of Pisa, Milan, Insubria and the CNR helped in advertising the research sending information through their mailing lists and sharing the news on their social pages (Facebook and Instagram); we created a public page for the survey on both Facebook and Instagram, and we shared them on our personal social pages; news were also posted on our personal profiles on Twitter and Linkedin.

The study consisted of a questionnaire to be completed online. Informed consent and consent to the processing of personal data had to be accepted on the first page of the online questionnaire. If consent was refused the individuals were excluded from the study.

#### The survey

In order to record the health status of the participants, a composed questionnaire was designed. It comprised three validated questionnaires that are repeated for two times, one for the period preceding the general lock-down and one for the period preceding the survey administration (when there were no restrictions), and a set of socio-demographic questions needed to characterize the respondents.

In this way it was possible to compare the individual self-reported health status between the two periods with the aim of highlighting the possible effect of lock down on the individuals.

The questionnaire used for this survey was composed as follows:

- **Short Form 36 health survey questionnaire** (SF-36): it is a self administered questionnaire that evaluates people’s quality of life by 36 items. It measures individual’s health on eight multi-item dimensions, covering functional status (i.e., physiological status, social functioning, role limitations-physical, and role limitations-emotional), well being (i.e., mental health, vitality, and bodily pain), and overall evaluation of health^15,16^. Likert scales and yes/no options are used to assess function and well-being on this 36-item questionnaire. This questionnaire was developed from the Medical Outcomes Study in English^17–19^, while the Italian version was validated by Apolone *et al*.^20^.
- **Single-Item Sleep Quality Scale** (SQS): SQS is a sleep quality measure developed to provide a more pragmatic approach for the assessment of sleep quality. Respondents are asked to mark an integer score from 0 to 10, according to the following five categories: 0 = terrible, 1–3 = poor, 4–6 = fair, 7–9 = good, and 10 = excellent. When rating their sleep quality, respondents are instructed to consider the following core components of sleep quality: how many hours of sleep they had, how easily they fell asleep, how often they woke up during the night (except to go to the bathroom), how often they woke up earlier than they had to in the morning, and how refreshing their sleep was. Snyder and colleagues^21^ validate single-item SQS comparing its score with the Pittsburg Sleep Quality Index (PSQI). Actually, they detected a strong correlation between the two sleep quality scores (r = 0.92) demonstrating that a single-item score is sensible as lengthier or more frequently administered sleep questionnaires^21^.
- **International Physical Activity Questionnaires** (IPAQ): IPAQ was developed as a surveillance tool to measure multiple domains of physical activity and inactivity^22^. In this questionnaire the short-version was used. The short form records is composed by 9 items evaluating the respondents activities in four intensity levels: 1) vigorous-intensity activity such as aerobics, 2) moderate-intensity activity such as leisure cycling, 3) walking, and 4) sitting. Italian validation was provided by Mannocchi *et al*.^23^.

To guarantee the anonymity of the data, personal information that permitted to re-identify the subjects involved in the study were not included (e.g. name and surname). Information needed to possibly stratify the participants included: i) gender; ii) age; iii) height; iv) weight; v) area of residence; vi) housing information; vii) working information; viii) sports participation; ix) Covid-related questions.

**Fig. 1:**
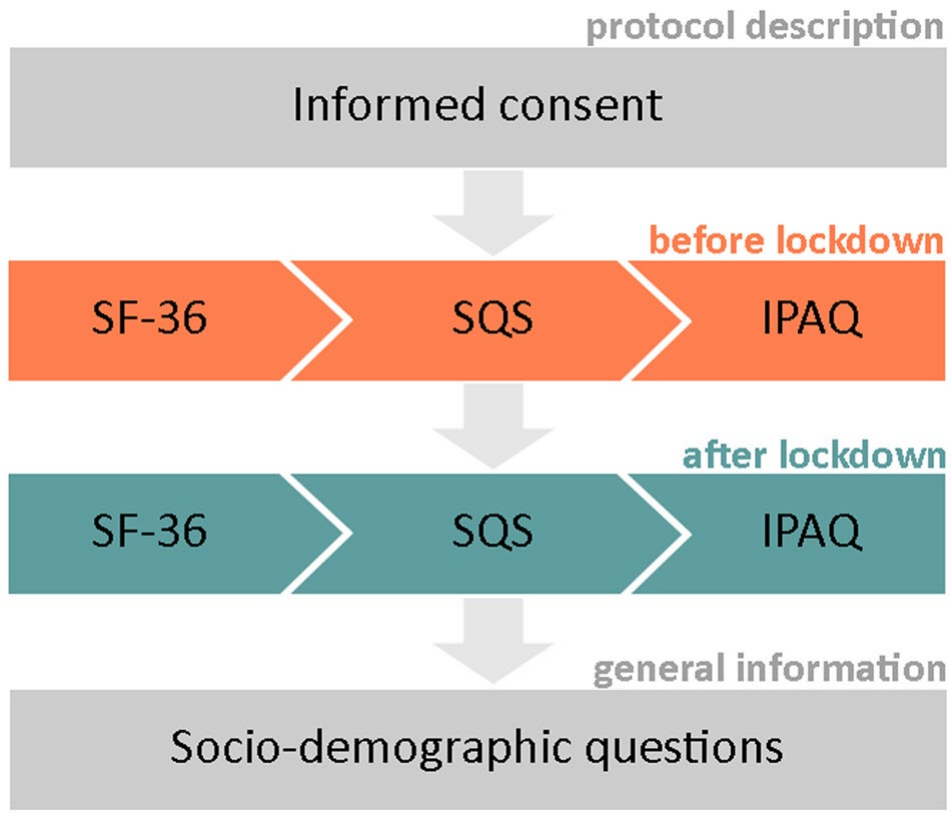
Questionnaire structure.

The questionnaire structure is described in Fig. 1.

## Data Records

The data sets are released under the CC BY 4.0 License and are publicly available on Figshare^24^. In the Figshare repository, two files are stored:

1. a *csv* file with the participants’ answers (data.csv);
2. a *json* file with the description of all the questions (columns) asked in the questionnaire (questions_answers_eng.json).

Table 1 reports an example of the dataset provided in data.csv. Each row in the dataset refers to a single respondent, while the columns refer to the answers for each question in the dataset. The questions_answers_eng.json file provides information about each question. Box 1 provides an example of a document that describes a specific columns in the dataset. The key of each document in the json file refers to the column name in the dataset. In each document there are four keys:

- question: string with the question asked in the questionnaire.
- answers: information about all the possible answers to the question. Two type of values are possible: i) string describing the type of data, i.e. “multiple answer”, “continuous variable”, “categorical variable”; ii) a dictionary useful to map the code in the dataset. In details:

**Table 1:** Dataset example.

ResponsedId	Q4	Q6_1	Q6_2	Q6_3	Q6_4	Q6_5	…	Q74_1	Q75.1	Q76	Q77	zone
R_3m4USzYaeCi7lsN	2	1	2	2	2	2	…	5	0	0	2	0
R_2dFjcJsDD66ipfz	3	2	2	2	2	2	…	3	0	0	2	0
…	…	…	…	…	…	…	…	…	…	…	…	…
R_3ebqylKLwbHGjnD	4	2	2	2	2	2	…	4	0	1	1	1
R_3rPqvckvP6Hjbjz	2	1	1	1	1	1	…	4	0	0	2	2

- Multiple answer: when a question could have multiple choice (e.g., “Which sporting activity do you practice?”)

- Continuous variable: when the question permits a numerical answer (e.g., the year of birth, the weight and the height)

- Categorical variable: when a question can take as answer one possible category (e.g., “In which region do you live?”)

In the specific case in which the answers are categorical but require a mapping to decode the answer, a dictionary is provided. In this dictionary the key represents the numerical code derived from the questionnaire, while the value represents the respective codification.

For example, in Box 1 for the “answers” key, the value is a dictionary in which the key = 0 stands for “No” while key = 1 means “Yes”. In this way it is possible to map the code for this specific question in the dataset.

- type: information about the type of question. The possible values are: “IPAQ”, “SF-36”, “SQS”, “comparison”, “respondent characteristics”. IPAQ (International Physical Activity Questionnaires), SF-36 (Short Form 36 health survey questionnaire), and SQS (Single-Item Sleep Quality Scale) refer to the specific sub-questionnaire (as described before). “Comparison” reflects questions that compares a specific status before and after the lockdown, while “respondent characteristics” refers to all the questions that permit to stratify the respondents in accordance with their individuals’ characteristics (e.g., gender, age, and region of residence).
- period: information about the period that referring the question. The possible values are: “before lockdown”, “during”, “after lockdown”, “comparison”, “general”. “Before lockdown”, “during” and “after lockdown”, refer to a specific period that the answer referred to. “Comparison” reflects questions that compares a specific health status before and after the lockdown, while “general” refers to question that did not refer to a specific period such as, for example, the gender or region of residence of the respondents.

Personal information that permits to stratify the sample was asked at the end of the questionnaire. In particular, the individuals’ information recorded are: gender, year of birth, weight, height, Italian zone where the respondent live and the respondents’ work characteristics (sedentary or active). Moreover, information about COVID-19 contraction was also asked at the end of the questionnaire.

Box 1 Example of a document in the questions_answers_eng.json file.

“Q76”: “question”: {“Has the pandemic changed the way you perceive the importance of practicing sports/physical activity regularly?”,“answers”: {0: “No”, 1: “Yes”},“type”: “comparison”,“period”: “comparison”}

## Technical Validation

### Data preparation

**Fig. 2:**
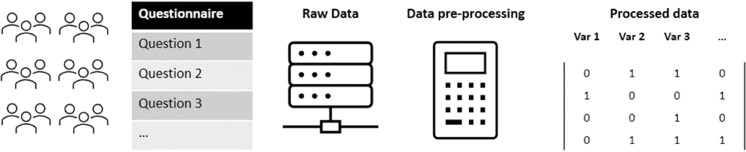
Data pre-processing steps.

The data was retrieved from the Qualtrics® Survey Platform in csv format. Any information that the software collects (e.g. geo-location) was deleted. The data, in csv format was then analyzed. A Python script was written to assess the quality of the data collected and to verify that all attributes were within the expected ranges. We calculated descriptive statistics for all the variables to verify the presence of implausible values and outliers. Many of the questions were Likert-type scales, with a predefined number of response options (e.g., 0 to 6). For these questions, we verified that they were numeric and suitable for subsequent transformations. Impossible values were replaced with missing values (e.g. height greater than 300 cm, weight lower than 20 Kg). Records with more than 30. The entire data acquisition and pre-processing is shown in Fig. 2.

Given the structure and characteristics of the questionnaires, we prepared in the Python code^24^ to calculate the aggregated indicators that are helpful in measuring the different dimensions that compose the general state of well-being^15^, sleep quality^21^ and physical activity^22^. Starting from the SF-36, we derived 8 multi-item indicators: i) physiological functioning, ii) social functioning, iii) role limitations due to physical health, iv) role limitations due to emotional problem, v) emotional well-being, vi) energy/fatigue, vii) bodily pain, and viii) general health. These dimensions are all scored between 0 and 100, where 0 indicates low health and 100 indicates high health. Additionally, the IPAQ assessed physical activity habits and sedentary behaviors. The IPAQ aggregate indicator assesses the types and the intensity of the physical activity providing the total energy expenditure as MET (Metabolic Equivalent of Task) per week. Furthermore, it was possible to calculate an estimation of sedentary behaviors by assessing the sitting time during week and weekend days. Finally, the sleep quality was obtained using a single-item question called SQS (10-points Likert scale, where 0 and 10 indicate terrible and excellent sleep quality, respectively).

### Participants’ stratification

2328 Italians took part in this study. Table 2 shows the distribution of respondents’ characteristics. In particular, in this dataset, about 62% of the respondents are female, 65% are young adult, i.e. aged between 18 and 30 years old, and, in accordance with Body Mass Index (BMI), 52% are normal weight. Moreover, the highest number of respondents come from the centre of Italy (75%), while only 20% and 5% of the respondents live in North and South Italy, respectively. 63% of the respondents have a sedentary job, while the 17% and 20% of the participants have mixed and active occupations, respectively. Furthermore, during the lockdown, the large majority of the respondents (71%) lived in a house with an external space large enough to do some physical activity. Finally, the greater part of the respondents neither got COVID-19 nor have encountered any related symptoms (90.04%). Only 9.53%, 0.39%, and 0.05% showed mild, moderate and severe symptoms, respectively.

**Table 2:** Description of respondents’ characteristics.

Characteristic	Group	Frequency
Gender	Male	863 (37.07%)
	Female	1449 (62.24%)
	Not defined	16 (0.69%)
Age	18–30	1518 (65.21%)
	31–40	247 (10.61%)
	40–50	195 (8.38%)
	>50	368 (15.81%)
BMI	Underweight	500 (21.48%)
	Normalweight	1210 (51.98%)
	Overweight	618 (26.55%)
Residence Area	North	462 (19.85%)
	Centre	1738 (74.65%)
	South	128 (5.50%)
House during lockdown	External space	1650 (70.88%)
	No external space	678 (29.12%)
Work type	Sedentary	407 (35.21%)
	Moderate Sedentary	316 (27.34%)
	Mixed	205 (17.73%)
	Moderate Active	115 (9.95%)
	Active	113 (9.78%)
COVID	No	2096 (90.03%)
	Mild symptoms	222 (9.54%)
	Moderate symptom	9 (0.39%)
	Severe symptom	1 (0.04%)

### Health perception

A decrease in the perceived quality of life, mental health and well-being was observed in previous studies during the lockdown in healthy elderly persons^12^, Brazilian population^25^, and Italian adults^14,26^. Actually, Bordeur and colleagues^13^ reported a substantial increase in the online search intensity for topics related to boredom, loneliness, worry, and sadness, and a substantial reduction of users’ interest about stress, suicide, and divorce topics in Europe and the US. These results suggest that people’s mental health may have been severely affected by the pandemic and lockdown. Moreover, despite people perceived a gradual return to their regular life immediately after the end of lockdown, detrimental health consequences of the COVID-19 pandemics still persisted several months later^27–29^.

**Fig. 3:**
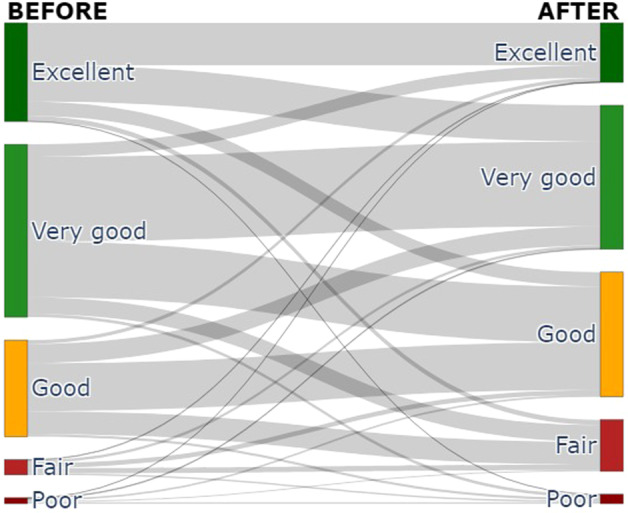
Sankey diagram describing the change in perceived quality of life.

In this dataset, 45.89% of respondents did not perceive any change in health. However, the health status of 45.20% of our respondents got worse, while only 8.91% of them perceived an increase in their health status. This result is corroborated by the sankey diagram provided in Fig. 3. In this Figure it is possible to observe that the 10.72% of the people changed from a positive health perception (i.e., excellent, very good, and good) to a negative one (i.e., fair and poor). In particular, the responses frequency in excellent and very good health classes were reduced of about 66% and 17% after the lockdown, respectively, while good, fair, and poor answers were increased of about 20%, 70%, and 50%, respectively. The results of each personal dimension evaluated by using the SF-36 questionnaire provided in Table 3 corroborate this result. As a matter of fact, the reduction of scores in almost all of the quality of life’s domains after the lockdown period suggests a worsening of the respondents’ health status.

**Table 3:** Questionnaire results. The scores are reported as mean ± standard deviation.

Questionnaire	Dimension	Before lockdown	After lockdown
SF-36	Physical functioning	95.84 ± 11.55	92.82 ± 12.79
	Role limitations-physical	91.15 ± 22.45	72.21 ± 36.10
	Role limitations-emotional	78.82 ± 34.68	48.82 ± 44.29
	Energy/fatigue	55.13 ± 10.63	55.42 ± 11.75
	Emotional well-being	67.19 ± 17.51	58.06 ± 21.09
	Social functioning	77.01 ± 21.09	63.03 ± 27.78
	Bodily pain	91.99 ± 15.85	81.53 ± 21.89
	General health perception	91.99 ± 15.85	64.75 ± 18.43
SQS	Sleep quality	7.07 ± 1.79	6.34 ± 1.99
IPAQ	Energy Expenditure (METs per week)	3462.05 ± 3713.13	2841.02 ± 3515.27
	Sedentary time during week (minutes)	350 ± 183	393 ± 197
	Sedentary time during weekend (minutes)	227 ± 162	271 ± 188

### Sleep quality

COVID-19 outbreak had a severe impact on people’s sleep quality. Different changes in sleep behaviours were identified by previous studies^7–10^ during lockdown. As a matter of fact, three main changes in sleep patterns have emerged from these studies. Some people have reduced the time spent in bed showing a significant shortening of total sleep time, a difficulty to maintain sleep, insomnia, and morning awakenings. The second group have increased their bed time yet maintaining their usual sleep patterns, while the third group increased their bed time but had problems falling asleep thus experiencing long sleep-onset latency, sleep initiations and maintenance difficulties, insomnia, and early morning awakenings compared to the period before the COVID-19 lockdown. In addiction, a large part of the population shifted the sleep schedule later during lockdown compared to their preferred schedule. This change in sleep patterns could either affect and be affected by mental and physical health. First, stress disorders induced by movement restriction, and maladaptive coping strategies (e.g., elevated alcohol consumption and time watching television or small screens such as smartphone before sleeping), have been associated with sleep problems^7^. On the other hand, prolonged periods of irregular sleep routines might affect the sleep-wake circadian rhythmicity, thus causing an impoverishment of the sleep quality inducing an increased risk of mental and physical health disorders^30,31^. In this dataset, the sleep quality of the respondents does not seem to have returned to the values before the lockdown even after spending a long period of regular daily routine. As a matter of fact, low SQS score (poor sleep quality) was recorded after the lockdown period compared to the period before, when the respondents perceived a better sleep quality (Table 3). This dataset could help in detecting the reasons behind the reduction of sleep quality highlighting the consequences on Italians’ health status.

### Physical activity and sedentary behaviours

Regular exercise and physical activity positively influence all aspects of health, both physical and mental^32^. The movement restriction caused by COVID-19 lockdown have limited the possibility of people around the world to perform physical activity increasing their inactivity tendency and consequently the sedentary behaviours. Previous studies found a significant decrements in energy expenditure during lockdown compared to the pre-lockdown in several countries and populations around the world^11,26,33–45^.

**Fig. 4:**
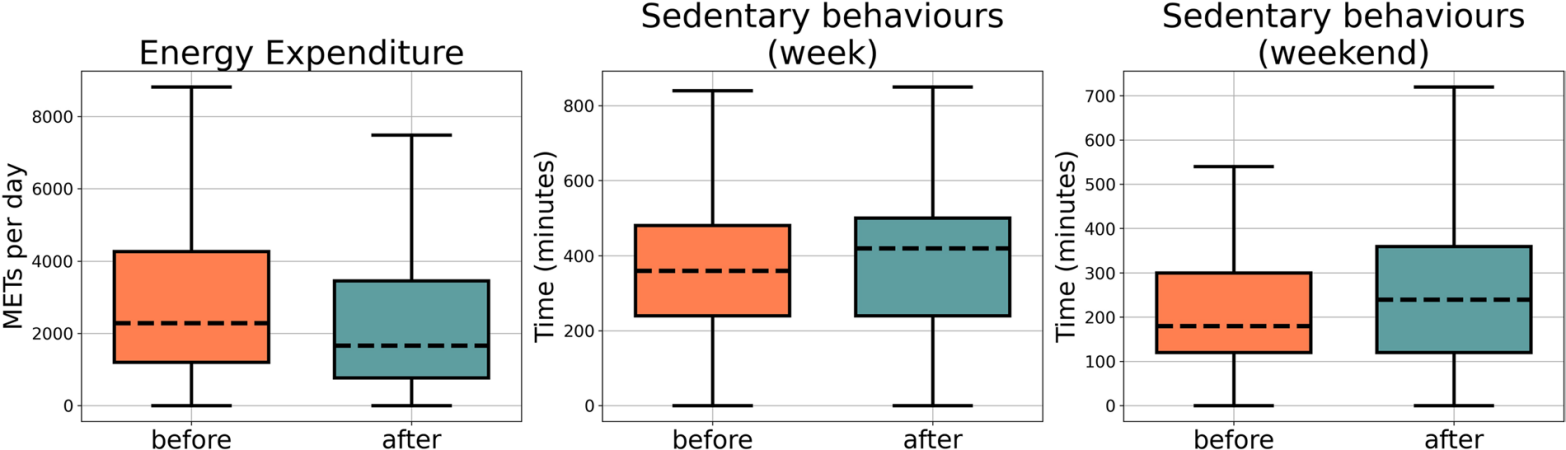
Boxplot of IPAQ scores.

Engaging in a sufficient amount of physical activity permits to reduce the risks of all-cause mortality having a particular effect on reducing cardiovascular problem, type 2 diabetes, cognitive dysfunction, and anxiety symptoms that negatively affect the people’s quality of life and physical functions^46^. The respondents of this survey seem to continue having sedentary behaviours also after several months from the end of the lockdown when compared to the pre-lockdown period. Indeed, Table 3 and Fig. 4 show that the metabolic equivalent of task per week (METs) are still lower compared to the period before the lockdown, despite the return to regular habits. Consequently, the sitting time increased from before to after lockdown period in both week and weekend days.

**Fig. 5:**
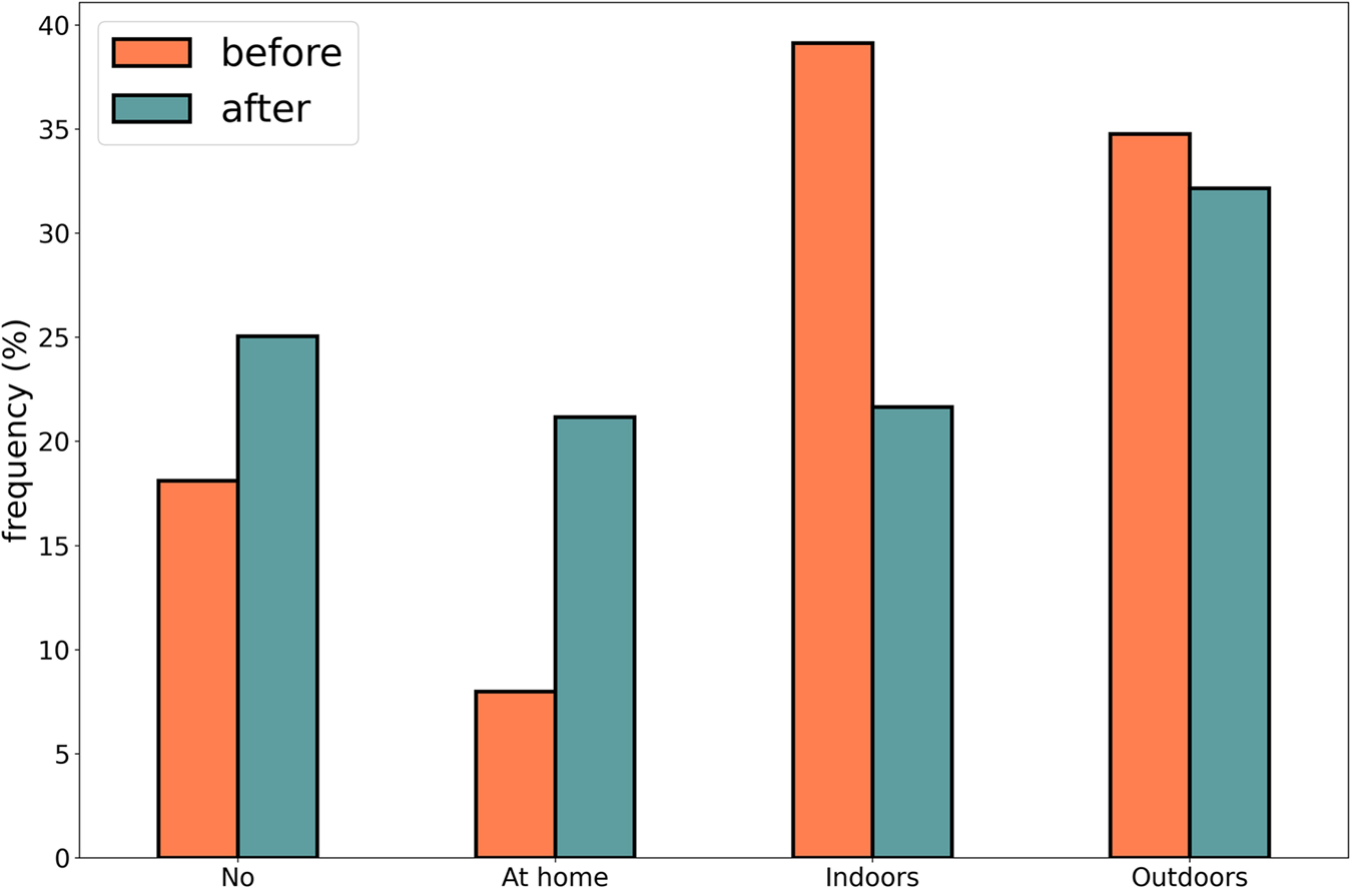
Distribution of physical activity practice before and after the lockdown period.

**Fig. 6:**
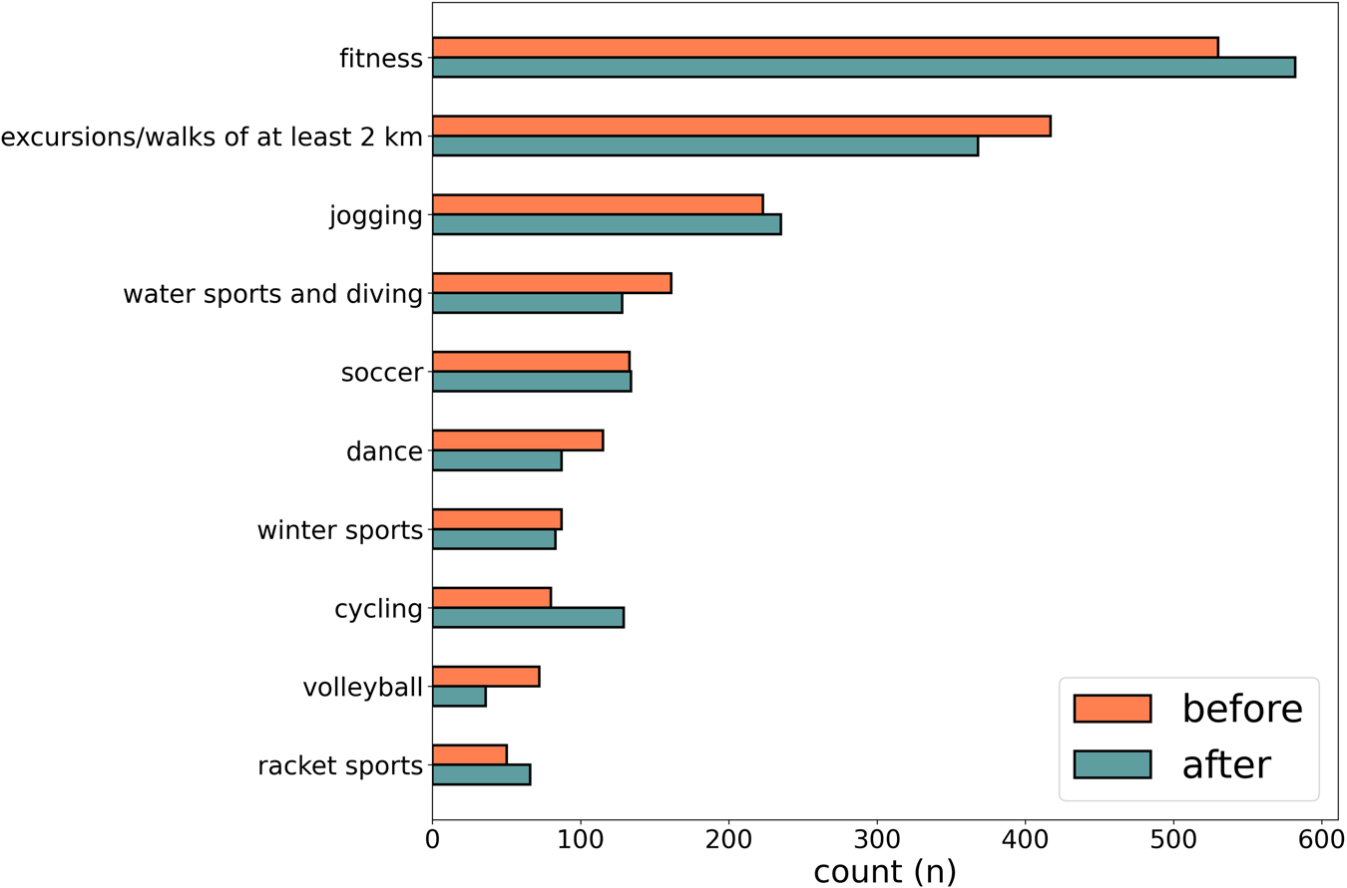
Distribution of sports practice before and after the lockdown period.

Among the respondents, 60% reported that their perception related to the importance of regularly practicing sports and physical activity was negatively changed. Actually, the sport disciplines performed by Italians slightly changed after lockdown. As shown in Fig. 5, the percentage of people who has not practiced any sports or physical activity increased of about 7% (from 18% recorded before lockdown to 25% observed after it). Moreover, Italians that regularly performed physical activity changed the location where they do sport. Figure 5 also shows that the percentage of people performing physical activity at home increased of about 13%, while the percentage of people performing physical activity indoors (e.g., fitness centres and swimming pools) and outdoors (e.g., parks and outdoor playgrounds) decreased of about 17% and 3%, respectively. The kind of sports practiced by Italians has changed as well. Figure 6 shows a high increment in fitness sports, jogging, and cycling, while a reduction was detected for excursions, water sports, dance, and volleyball. Future analysis based on this dataset will provide detailed insight about the change in physical activity habits, sedentary behaviours, and sports practice which could help expert to promote physical activity with the purpose of increasing people’s wellness status.

## Usage Notes

This dataset may be useful for investigating the impact of the COVID-19 lockdown on psychological and behavioural aspects in Italians. In particular, it will be possible to assess whether the quality of life, sleep, and physical activities could return to pre-isolation levels after a sufficient period without movement restrictions (from May 2021 to November/December 2021). The detailed information on each aspect of the respondents’ life will permit to have a complete overview of their habits, perceptions, and well-being status before and after the COVID-19 lockdown. Moreover, this dataset can give accurate insights on the changes in the population general health status: these analyses will provide practical suggestion at local, regional, and national level to improve infrastructures and services, thus improving Italians’ well being.

Furthermore, despite the many benefits due to the vaccination campaign in reducing the transmissibility of the virus, the presence of several COVID-19 variants does not make us safe with respect to possible future actions^47^: understanding the consequences of the restrictions may help to better design strategies to contain virus widespread and, at the same time, to ensure a reduced impact on the lives of citizens in terms of well-being, sleep quality, and physical activities.

Finally, as this dataset is related to public health, the benefits of having it open to other researchers are many: (i) to reduce the cost of public research, avoiding the need of launching new studies in this field^48^; (ii) to replicate/repeat experiments in a trustworthy way, perhaps with somewhat different methods^49^; to increase the trust of public opinion toward science, and to encourage ‘data altruism’ with citizens to facilitate data sharing^50^.

It should be noted, in accordance with the study design, that the information about the pre-lockdown period could be affected by a memory-related issue. In fact, in the questions related to the period before the restrictions, the respondents were requested to remember details about their life of more than 2 years before (the so called memory effect^51^). When adding retrospective questions in a survey, respondents tend to give less accurate answers since these questions place high cognitive demands on themselves^52^. This recall bias can be crucial especially when, as in our case, the period between the data collection and the pre-lockdown is quite large. On the other hand, using specific anchor points, as done in this survey, can help respondents to remember their health status and other items of interest^53^ and, on aggregate level the results tend to be less unreliable than at an individual level^52^. Thus, in analyzing these data, one must be aware of the possible presence of recall bias, and to keep in mind that comparisons between before and after lockdown are more reliable at the aggregate level. Hence, the comparison of the before and after lockdown periods reflects the perception of change between the two time periods.

Thanks to some additional information of the respondents such as socioeconomic status, living space, and employment, it will be possible to stratify the sample with the aim of assessing the COVID-19 lockdown effect on specific population subgroups. In this way, it will be possible to obtain insights about a specific part of the population suggesting *ad hoc* interventions. To be noticed, the number of respondents per geographical area is not balanced, so this dataset does not allow appropriate geographical comparisons.

Last but not least, this dataset not only could be used for understanding changes in Italians’ habits with a public health purpose but could be a starting point for several other research fields. For example, it could be possible to evaluate if there are some alterations in shopping habits and Google search intents of Italians after the lockdown in relation with the changes in physical activity habits, sleep patterns, and life quality with a marketing purpose.

## Data Availability

The code to reproduce the plots in the paper is publicly available on Figshare^24^.
